# P-2109. Infrequent PJP Prophylaxis in Non-HIV, Non-Transplant Adults Receiving Immunosuppressants Diagnosed with PJP

**DOI:** 10.1093/ofid/ofaf695.2273

**Published:** 2026-01-11

**Authors:** Alexander J Ruehman, Melat Endashaw, Andrés F Henao Martínez, Alfonso G Bastias, Brian T Montague, Daniel B Chastain

**Affiliations:** University of Maryland Medical Center, Baltimore, MD; Emory Healthcare, Atlanta, GA; University of Colorado Anschutz Medical Campus, Aurora, Colorado; University of Colorado Boulder, Boulder, Colorado; University of Colorado School of Medicine, Arvada, Colorado; University of Georgia College of Pharmacy, Albany, GA

## Abstract

**Background:**

*Pneumocystis jirovecii* pneumonia (PJP) is increasingly recognized in non-HIV populations. However, prophylaxis in non-HIV, non-transplant adults receiving immunosuppressants remains inconsistent due to heterogeneous risk profiles. The cumulative impact of comorbidities and immunosuppressants on PJP susceptibility in this growing population is not well-defined. We examined baseline characteristics, immunosuppressant use, and PJP prophylaxis rates in non-HIV, non-transplant adults with PJP.

**Table:**
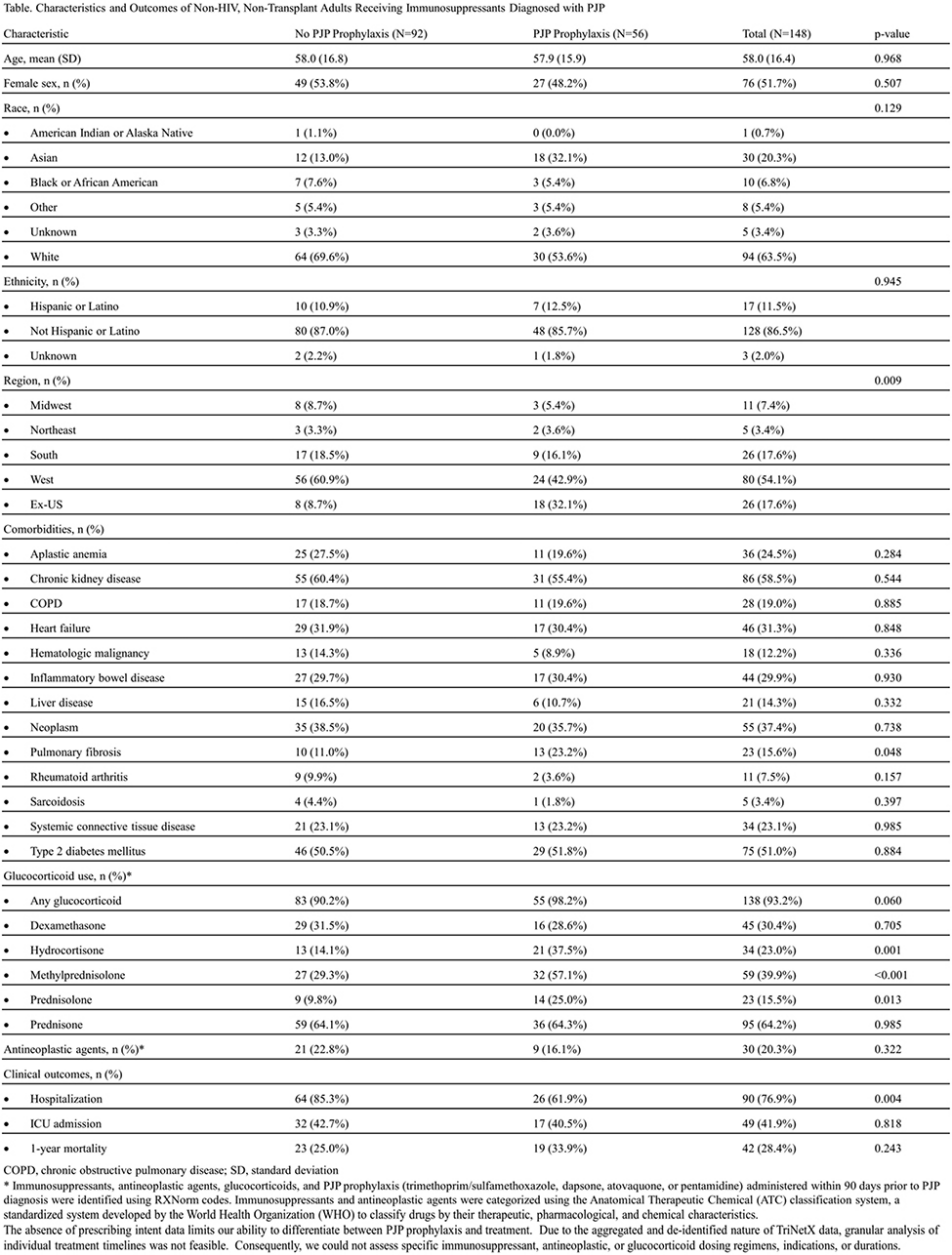
Characteristics and Outcomes of Non-HIV, Non-Transplant Adults Receiving Immunosuppressants Diagnosed with PJP

**Methods:**

We conducted a retrospective cohort study using TriNetX, a federated network of anonymized health records from > 250 million patients across 250 healthcare organizations in 30 countries. Adults (≥ 18 years) on immunosuppressants diagnosed with PJP (positive PCR or antigen test) between 2011 and 2024 were included if they had no history of HIV or solid organ transplantation. We analyzed demographics, comorbidities, concurrent antineoplastic agents and glucocorticoids, PJP prophylaxis (trimethoprim/sulfamethoxazole, dapsone, atovaquone, or pentamidine prescriptions within 90 days pre-diagnosis), hospitalizations, including ICU admissions, and one-year mortality.

**Results:**

Of the 148 patients, only 38% received prophylaxis (Table). Demographics were similar between groups, with higher prophylaxis rates outside the US and lower rates within the US (p=0.009). Most comorbid conditions were comparable, but pulmonary fibrosis was more common in the prophylaxis group (23% vs 11%, p=0.048). The types of immunosuppressant and antineoplastic agents were comparable between groups. Although overall glucocorticoid use was similar, the use of hydrocortisone, methylprednisolone, and prednisolone was significantly more frequent in patients who received prophylaxis (p≤ 0.013). Hospitalization was less common in the prophylaxis group (62% vs 85%, p=0.004), while ICU admission and one-year mortality rates were comparable.

**Conclusion:**

PJP prophylaxis was infrequently prescribed among non-HIV, non-transplant adults on immunosuppressants diagnosed with PJP. The similar age, sex distribution, prevalence of many comorbidities, and types of immunosuppressants across groups suggest that PJP prophylaxis decisions were not consistently guided by these factors.

**Disclosures:**

Andrés F. Henao Martínez, MD, MPH, F2: Grant/Research Support|Scynexis: Grant/Research Support

